# Supplementary material for: Mutations in the non-catalytic polyproline motif destabilize TREX1 and amplify cGAS-STING signaling
Source: bioRxiv. 2024 Jan 4:2024.01.04.574136. Preprint. [Version 1] doi: 10.1101/2024.01.04.574136 (PMC10802300; doi:10.1101/2024.01.04.574136)
Supplement: Supplement 1 [file NIHPP2024.01.04.574136v1-supplement-1.pdf]

bioRxiv preprint doi: https://doi.org/10.1101/2024.01.04.574136; this version posted January 4, 2024. The copyright holder for this preprint (which was not certified by peer review) is the author/funder, who has granted bioRxiv a license to display the preprint in perpetuity. It is made available under aCC-BY 4.0 International license.

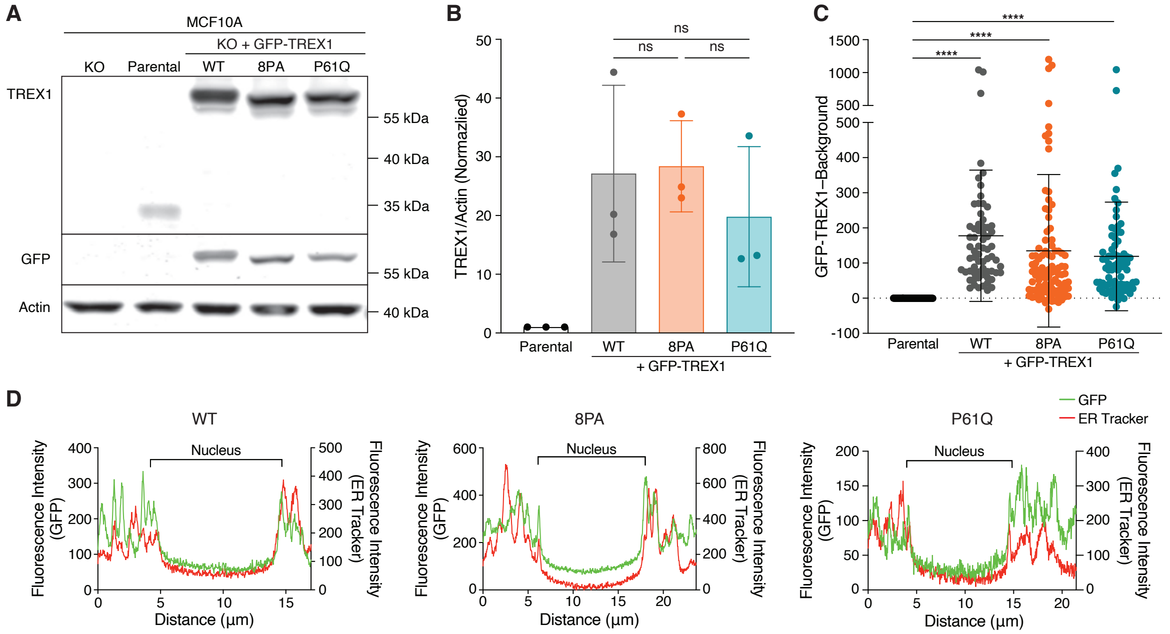

Figure S1. Overexpressed TREX1 mutants localize to the ER. A. Representative immunoblot of MCF10A *TREX1* KO cells reconstituted with the indicated GFP-TREX1, using anti-TREX1, anti-GFP, and anti-actin antibodies. **B.** Quantification of TREX1 immunoblot signal normalized to actin; mean ± s.d., $n = 3$, ns = not significant, one-way ANOVA ($p = 0.0401$). For each replicate, the parental TREX1/Actin signal was set to one. **C.** Quantification of GFP-TREX1 signal in the indicated cells as in Fig. 1G; mean ± s.d., $n = 5$ experiments, ****$p < 0.0001$, one-way ANOVA ($p < 0.0001$).**D.** Line profile analysis of the indicated cells as in Fig. 1G. Position of the nucleus was determined using the line profile signal of the DAPI channel. Background signal was subtracted from all points.

bioRxiv preprint doi: https://doi.org/10.1101/2024.01.04.574136; this version posted January 4, 2024. The copyright holder for this preprint (which was not certified by peer review) is the author/funder, who has granted bioRxiv a license to display the preprint in perpetuity. It is made available under aCC-BY 4.0 International license.

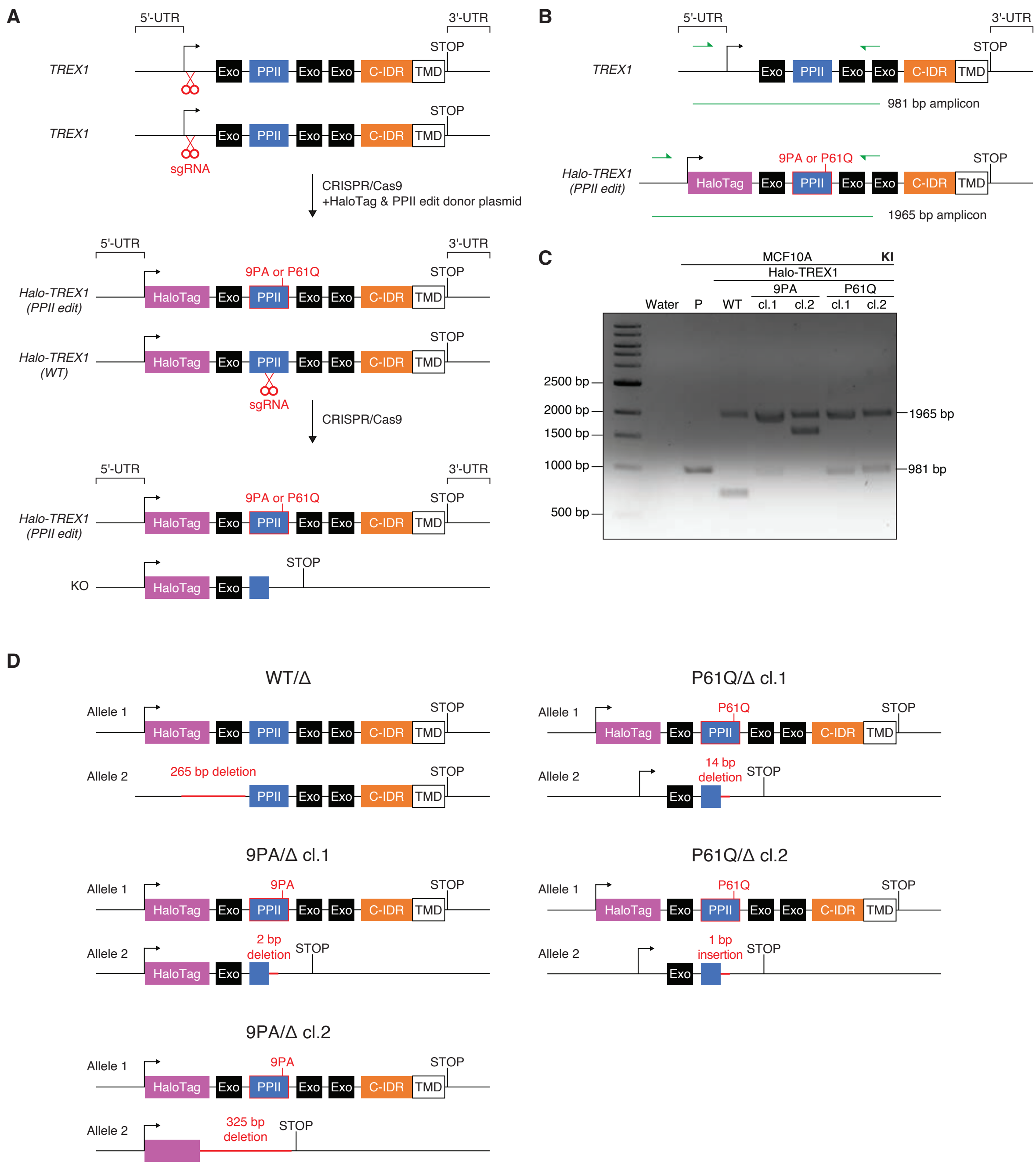

Figure S2. Generation of *TREX1* knock-in mutations. A. Representative schematic of *TREX1* gene editing protocol. Briefly, an N-terminal sgRNA and a HaloTag donor plasmid harboring a PPII edit in its downstream homology arm were nucleofected into MCF10A cells. Sanger sequencing revealed that HaloTag insertion occurs more frequently than incorporation of the PPII edit, often yielding two Halo-tagged alleles, one with the desired PPII edit. A second round

bioRxiv preprint doi: https://doi.org/10.1101/2024.01.04.574136; this version posted January 4, 2024. The copyright holder for this preprint (which was not certified by peer review) is the author/funder, who has granted bioRxiv a license to display the preprint in perpetuity. It is made available under aCC-BY 4.0 International license.

of gene editing was carried out using a sgRNA specific for unedited PPII, knocking out the unedited allele while leaving the PPII-edited, Halo-tagged allele intact. **B.** Schematic of PCR primers and amplicons used to validate knock-in cell lines. **C.** PCR gel of all knock-in cell line clones used in this manuscript. All bands were excised and Sanger sequenced to validate expected gene edits. **D.** Schematic detailing the precise edits present in all clones.

bioRxiv preprint doi: https://doi.org/10.1101/2024.01.04.574136; this version posted January 4, 2024. The copyright holder for this preprint (which was not certified by peer review) is the author/funder, who has granted bioRxiv a license to display the preprint in perpetuity. It is made available under aCC-BY 4.0 International license.

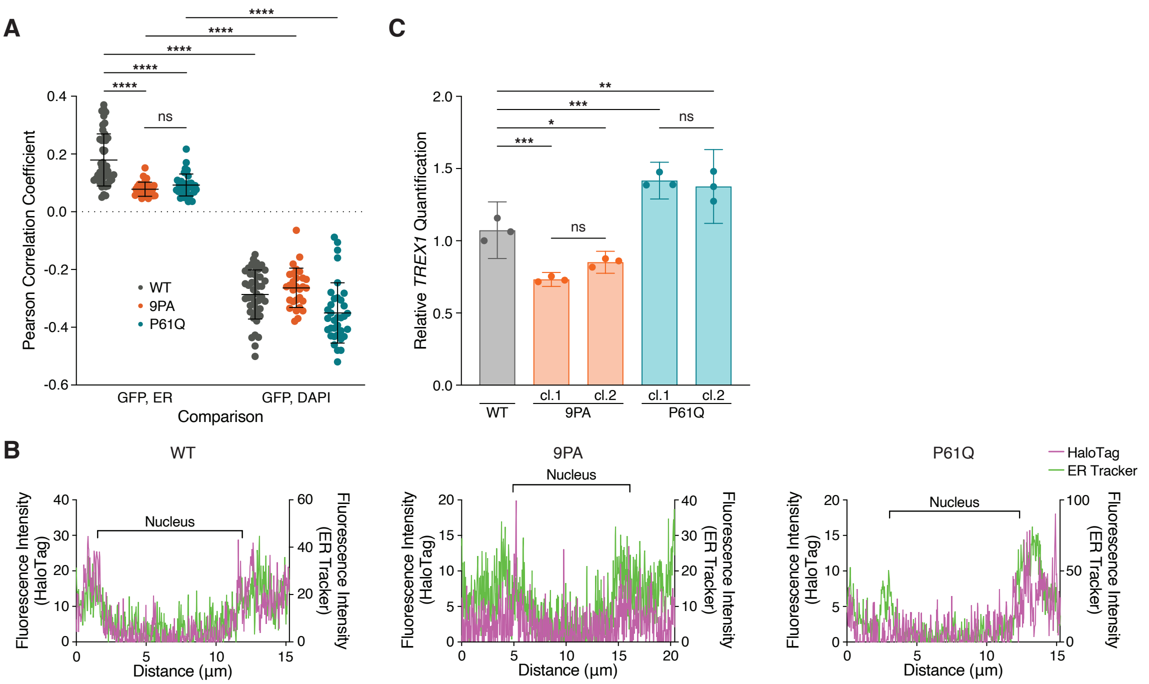

Figure S3. Mutations in PPII do not interfere with TREX1 transcription or localization. A. Pearson correlation coefficients of the indicated cells as in Fig. 3C; mean ± s.d., $n = 5$ experiments, ****$p < 0.0001$, ns = not significant, two-way ANOVA (interaction $p < 0.0001$, comparison pair $p < 0.0001$, genotype $p < 0.0001$). **B.** Line profile analysis as indicated in Fig. 3C. Extent of the nucleus was determined using the line profile signal of the DAPI channel. Background signal was subtracted from all points. **C.** RT-qPCR of *TREX1* in the indicated cells following mock transfection; mean ± s.d., $n = 3$, *$p < 0.05$, **$p < 0.01$, ***$p < 0.001$, ns = not significant, one-way ANOVA ($p < 0.0001$).
